# Epithelial Cell Stretching and Luminal Acidification Lead to a Retarded Development of Stria Vascularis and Deafness in Mice Lacking Pendrin

**DOI:** 10.1371/journal.pone.0017949

**Published:** 2011-03-14

**Authors:** Hyoung-Mi Kim, Philine Wangemann

**Affiliations:** Anatomy and Physiology Department, Kansas State University, Manhattan, Kansas, United States of America; Instituto Nacional de Câncer, Brazil

## Abstract

Loss-of-function mutations of *SLC26A4*/pendrin are among the most prevalent causes of deafness. Deafness and vestibular dysfunction in the corresponding mouse model, *Slc26a4^−/−^*, are associated with an enlargement and acidification of the membranous labyrinth. Here we relate the onset of expression of the HCO_3_^−^ transporter pendrin to the luminal pH and to enlargement-associated epithelial cell stretching. We determined expression with immunocytochemistry, cell stretching by digital morphometry and pH with double-barreled ion-selective electrodes. Pendrin was first expressed in the endolymphatic sac at embryonic day (E) 11.5, in the cochlear hook-region at E13.5, in the utricle and saccule at E14.5, in ampullae at E16.5, and in the upper turn of the cochlea at E17.5. Epithelial cell stretching in *Slc26a4^−/−^* mice began at E14.5. pH changes occurred first in the cochlea at E15.5 and in the endolymphatic sac at E17.5. At postnatal day 2, stria vascularis, outer sulcus and Reissner's membrane epithelial cells, and utricular and saccular transitional cells were stretched, whereas sensory cells in the cochlea, utricle and saccule did not differ between *Slc26a4^+/−^* and *Slc26a4^−/−^* mice. Structural development of stria vascularis, including vascularization, was retarded in *Slc26a4^−/−^* mice. In conclusion, the data demonstrate that the enlargement and stretching of non-sensory epithelial cells precedes luminal acidification in the cochlea and the endolymphatic sac. Stretching and luminal acidification may alter cell-to-cell communication and lead to the observed retarded development of stria vascularis, which may be an important step on the path to deafness in *Slc26a4^−/−^* mice, and possibly in humans, lacking functional pendrin expression.

## Introduction

Mutations of *SLC26A4* are found in some populations in as many as 13.7% of deaf subjects and are thereby among the most prevalent causes of deafness [1], [2], [3], [4], [5]. Phenotypes associated with mutations of *SLC26A4* include deafness at birth and fluctuating hearing loss that progresses toward deafness during childhood [6], [7], [8]. Deafness is associated with balance dysfunction in a subset of patients [9]. The high incidence of this disorder provides a strong imperative to investigate the etiology of the disease with the ultimate goal to develop strategies to preserve hearing in afflicted individuals.

The gene *SLC26A4* codes for the protein pendrin, which is an anion-exchanger that is expressed in apical membranes of inner ear epithelial cells and transports HCO_3_^−^ into the luminal fluid [10], [11]. Pendrin-mediated HCO_3_^−^ secretion is responsible for the pH of cochlear endolymph to be higher than the pH of perilymph, which is the fluid surrounding the cochlear duct.

Work toward an understanding of the role of pendrin in hearing and balance has been accelerated by a conventional knock out mouse model, *Slc26a4^−/−^*, formerly called *Pds^−/−^* [12]. Studies using this mouse model have revealed that key events in the etiology of deafness are an acidification and enlargement of the membranous labyrinth that includes a ∼10-fold enlargement of the cochlear lumen [12], [13], [14], [10]. The enlargement of the cochlea lumen begins at embryonic day (E) 14.5, which is ∼19 days before the onset of hearing at postnatal day (P) 12 [12], [13], [14]. The enlargement is the result of fluid secretion in the vestibular labyrinth that ‘pumps up’ the cochlea and a failure of fluid absorption in the endolymphatic sac that ‘drains’ the cochlea at this age [14]. The enlargement and the acidification spread the effect of lacking pendrin-expression from affecting solely pendrin-expressing cells themselves to affecting a multitude of other cells in the cochlea and vestibular labyrinth, thereby ultimately leading to a failure to develop proper hearing and vestibular function.

The question how lack of pendrin affects fluid transport in the embryonic inner ear is complicated by the fact that pendrin is expressed in multiple epithelial cell types that are located in small domains throughout the inner ear. Based on studies in adult mice, pendrin is expressed in the cochlea in outer sulcus and spiral prominence epithelial cells and in spindle cells of stria vascularis [15], [16]. Further, pendrin is expressed in the vestibular labyrinth in transitional cells of the saccule, utricle, and ampullae and in the mitochondria-rich cells of the endolymphatic sac [15], [16], [17]. The onset of pendrin expression and the onset of pH changes associated with a lack of functional pendrin are important toward an understanding of the role of pendrin in fluid transport. Thus, the first goal of the present study was to determine the onset of pendrin expression in different parts of the inner ear, including the cochlea, saccule, utricle, ampullae, and endolymphatic sac and to determine the onset of pH changes that occur in the cochlea and endolymphatic sac when functional pendrin expression is present or lacking.

Loss of functional pendrin expression leads to an enlargement of the membranous labyrinth that displaces mesenchymal cells that normally surround the epithelial duct. This displacement impairs mesenchymal-epithelial cell communication including cochlear thyroid hormone signaling, which is important for the early postnatal development of the cochlea. Disruption of cochlear thyroid hormone signaling in *Slc26a4^−/−^* mice leads to cochlear hypothyroidism in the early postnatal phase of development [13]. The enlargement of the cochlea lumen may not only disrupt communication between mesenchymal and epithelial cells but may also disrupt cell signaling between adjacent epithelial cells. Stretching of epithelial cells can be expected to lengthen diffusional distances, which would dampen or delay the arrival of diffusional signals. Normal cochlear development depends on cell-to-cell communication. Loss of cell-to-cell communication, i.e. due to lack of connexin 26 expression, leads to retarded development and failure to acquire hearing [18], [19], [20]. Impairment of cell signaling and disruption of proper orchestration of development may also be a cause for the failure to develop hearing and balance in mice and possibly in humans lacking pendrin expression. Thus, the second goal of this study was to determine the onset of epithelial cell stretching in the cochlea and the endolymphatic sac and to determine which epithelial cell types are stretched in the cochlea and the vestibular labyrinth of mice lacking functional pendrin expression.

## Methods

### Animals

A colony of *Slc26a4^−/−^* and *Slc26a4^+/−^* mice was maintained at Kansas State University. Pairs of *Slc26a4^+/−^* dams and *Slc26a4^−/−^* sires were housed together. Litters sizes averaged 5.1 pups with *Slc26a4^+/−^* and *Slc26a4^−/−^* offspring in the Mendelian ratio of 50.1 to 49.9. The gestational period was 21 days. Genetic drift was limited by occasional back-crossing to the original strain, 129SvEvTac, maintained by Taconic, Germantown, NY. The colony was maintained free of known and suspected murine pathogens. Serologic tests (Radil, Columbia, MO), that were consistently and repeatedly negative, included the following antibodies: EDIM (Epizootic diarrhea of infant mice virus - a mouse rotavirus), TMEV (Theiler's murine encephalomyelitis virus - mouse poliovirus, strain GDVII), MHV (Mouse hepatitis virus - a mouse coronavirus), MVM (Minute virus of mice - a mouse parvovirus), MNV (Murine norovirus - a mouse calicivirus), M. pulmonis (Mycoplasma pulmonis – the agent of murine mycoplasmosis), MPV (a mouse parvovirus), Parvo NS-1 (a conserved recombinant parvoviral protein, rNS1) and Sendai (Sendai virus – a type 1 paramyxovirus).

*Slc26a4^+/−^* and *Slc26a4^−/−^* mice ranging in age from embryonic (E) day 11.5 to postnatal (P) day 7 were used in the present study. Time-pregnant dams were deeply anesthetized with 4% tri-bromo-ethanol and sacrificed by decapitation after harvesting embryos by sterile laparotomy. Gestational age was counted from the day, when a vaginal plug was detected. This day was set to E0.5. Gestational age, however, was verified, and in rare cases corrected, by evaluating gross morphological features including limbs, digits, and appearance of the pinna and auditory meatus [21], [22]. Neonatal mice (P2–P3) were anesthetized by a combination of i.p. injection of 0.013 ml/g body weight of 4% tri-bromo-ethanol and rapid cooling on an ice-water slush. Older mice (P7) were anesthetized solely by i.p. injection of 0.013 ml/g body weight of 4% tri-bromo-ethanol. Embryos and postnatal mice were sacrificed by decapitation.

### Ethics Statement

All procedures involving animals were approved by the Institutional Animal Care and Use Committee of Kansas State University (IACUC#: 2613 and 2961).

### Immunocytochemistry

For whole-mounts, tissues were isolated by microdissection and fixed at 4°C for 2 hrs in a PBS-solution containing (in mM) 150 NaCl, 3.6 KCl, 5 HEPES, 1 CaCl_2_, 1 MgCl_2_ and 5 glucose, pH 7.4 and 4% paraformaldehyde. Whole-mounts were blocked for 1 hr with 5% bovine serum albumin (BSA) in PBS-TX solution containing (in mM) 137 NaCl, 2.7 KCl, 10.1 Na_2_HPO_4_, 1.8 KH_2_PO_4_, pH 7.4, and 0.2% Triton-X-100.

For cryo-sections, isolated otocysts from embryos and isolated inner ear from postnatal mice were fixed at 4°C for 2 hrs in a PBS-solution containing 4% paraformaldehyde. Fixed tissues were processed through a sucrose gradient (10% and 20%, each 20 min, followed by 30% overnight, all at 4°C), infiltrated with polyethylene glycol (Cat# 72592-B, Electron Microscopy Sciences, Hatfield, PA) and cryo-sectioned (12 µm, CM3050S, Leica, Germany). Serial sections throughout the entire cochlea were obtained embryos aged E11.5 and E14.5 and mid-modiolar sections of the cochlea were obtained from embryos between ages E15.5 and E18.5 and from neonates aged P2–P7. Sections were mounted on charged slides (Cat#22-230-900, Fisher) and blocked for 1 hr with 5% bovine serum albumin (BSA) in PBS-TX solution.

For immunocytochemistry, whole-mounts or sections were incubated overnight at 4°C with diluted primary antibody (1∶200 rabbit anti-pendrin, a gift from Dr. Søren Nielsen, Aarhus University, Denmark; 1∶200 rabbit-anti-Cx26 (Invitrogen, Carlsbad, CA) or 1∶200 rabbit anti-Na^+^/K^+^ ATPase alpha1 subunit (Novus Biologicals, Littleton, CO). Primary antibodies were diluted with PBS-TX containing 1–3% BSA. Whole-mounts or sections were washed three times in PBS-TX and incubated for 1 hr at room temperature with secondary antibody (Alexa594 conjugated goat-anti-rabbit (Invitrogen) diluted 1∶1,000 with PBS-TX containing 1–3% BSA. After washing three times in PBS-TX, whole-mounts or sections were incubated at room temperature for 20 min with phalloidin and 5 min with DAPI diluted with PBS-TX at 1∶40 and 1∶1,000, respectively. After staining, whole-mounts or sections were washed again three times with PBS-TX, cover-slipped with FluorSave (Calbiochem, La Jolla, CA) and observed by confocal laser scanning microscopy (LSM 510 Meta, Carl Zeiss, Göttingen, Germany).

Morphometric measurements including determinations of circumferences and cell surface areas were obtained using software provided with the confocal laser scanning microscope (Aim, LSM 510 Meta, Carl Zeiss). Observations reported are based on at least three independent observations.

### Electrophysiological pH measurements

The endolymphatic pH and the transepithelial potential were measured *in vitro* with double-barreled microelectrodes. Procedures were developed by modifying previously described protocols [23], [11]. Otocysts were isolated from embryonic mice aged E14.5–E17.5 in cold (4°C) dissection solution containing (mM): 150 NaCl, 4 KCl, 10 HEPES, 1.5 CaCl_2_, 1 MgCl_2_, and 5 glucose, pH 7.4. For pH measurements, otocysts were transferred into a bath chamber, stabilized with a suction pipette and superfused with warm (37°C) bicarbonate-solution containing (mM): 135 NaCl, 25 NaHCO_3_^−^, 4 KCl, 1.5 CaCl_2_, 1 MgCl_2_ and 5 glucose, bubbled with 5% CO_2_ 95% O_2_ to assume pH 7.3–7.4. Measurements in the cochlea were made via a round-window approach, which yields data from the hook-region of the cochlea. Measurements in the endolymphatic sac were made in the intermediate portion. Data were recorded digitally (DIGIDATA 1322A and AxoScope 9, Axon Instruments) and analyzed using custom software written by P.W. in LabTalk (Origin 6.0, The Origin Company, Northhampton, MA).

Calibration consisted of taking a reference value in the superfused bath solution and then obtaining the slope of the electrode in a perfused agar cup placed into the bath chamber. Agar cups holding ∼100 µl were prepared with warm (37°C) weakly-buffered Ringer solution. This method was devised to minimize the contribution of electrode drift and differences between reference electrodes. pH sensitive electrodes had a slope of 58±3 mV/pH unit (n = 40). Three calibration solutions with different pH values were used. Calibration solution contained (in mM): pH 6: 130 NaCl, 20 MES; pH 7: 130 NaCl, 20 HEPES; and pH 8: 130 NaCl, 20 tricine.

Double-barreled glass microelectrodes were manufactured from filament-containing glass tubing (World Precision Instruments 1B100F-4, Sarasota, FL) using a micropipette puller (Narishige PD-5, Tokyo, Japan). Prior to silanization, microelectrodes were baked at 180°C for 2 hrs to ensure dryness. The longer ion-selective barrel was mounted in the lid of a beaker. The beaker was heated to 210°C and silanized by a 90 s exposure to 0.02 ml dimethyldichlorosilane (Fluka 40136) at RT. The shorter reference barrel was protected from silanization by sealing the open end with Parafilm (Alcan Packaging, Chicago, IL). After silanization, microelectrodes were baked at 180°C for 3 hrs and tips were broken to a final O.D. of ∼3 µm. The reference barrel was filled with 1 M KCl and the ionselective barrel was filled at the tip with liquid ion exchanger (Hydrogen ionophore II - Cocktail A, Fluka 95297) and back-filled with buffer solution (500 mM KCl, 20 mM HEPES, pH 7.4).

### Statistics

Data are generally presented as average ± sem with N being the number of animals. In some instances averages are presented with SD, as indicated. Data acquired in paired experiments using littermates were evaluated by paired t-test. Significance was assumed when p<0.05.

## Results

### Onset of pendrin expression

**Figure 1:**
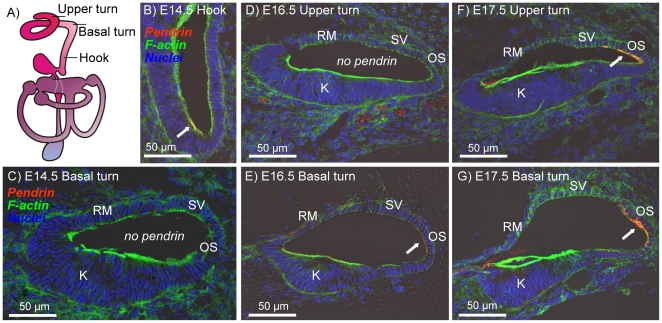
Onset of pendrin expression in the cochlea. Pendrin (*red*) was visualized by immunocytochemistry. F-actin (*green*) and nuclei (*blue*) were labeled. A: Diagram of the inner ear. B–G: Cross-sections of the cochlear duct in the hook region (B), basal turn (C, E and G) and upper turn (D and F) of *Slc26a4^+/−^* mice at age E14.5 (B and C), E16.5 (D and E) and E17.5 (F and G). Abbreviations: K, Kölliker's organ; RM, Reissner's membrane; SV, stria vascularis; OS, outer sulcus. Pendrin expression is marked by arrows.

In a first series of experiments, we determined the onset of pendrin expression in the embryonic cochlea, the vestibular labyrinth and the endolymphatic sac and duct. In the cochlea, pendrin expression was found in the outer sulcus region of the lateral wall. The onset of expression progressed from the hook region to the basal turn and then to the upper turn of the cochlea. The first expression was found at E13.5–E14.5 in the hook region of the cochlea (Fig. 1A–C). At this age, there was yet no expression in the basal turn. At E16.5, expression was found in the basal turn but not yet in the upper turn (Fig. 1D and E) and at E17.5 pendrin expression was found throughout the cochlea (Fig. 1F and G).

**Figure 2:**
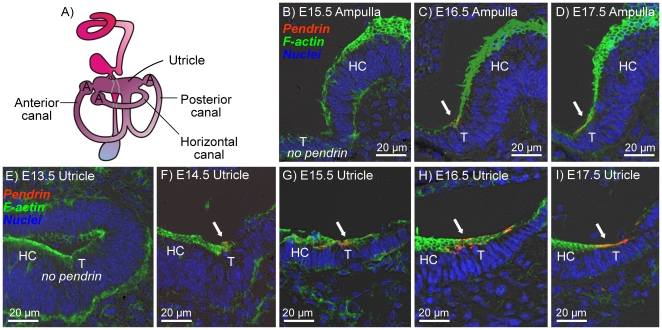
Onset of pendrin expression in the ampullae and utricle of the vestibular labyrinth. Pendrin (*red*) was visualized by immunocytochemistry. F-actin (*green*) and nuclei (*blue*) were labeled. A: Diagram of the inner ear. B–D: Cross-sections of the anterior canal ampulla of *Slc26a4^+/−^* mice at age E15.5–E17.5. E–I: Cross-sections of utricle of *Slc26a4^+/−^* mice at age E13.5–E17.5. Abbreviations: A, Ampulla; T, transitional cells; HC, hair cells. Pendrin expression is marked by arrows.

**Figure 3:**
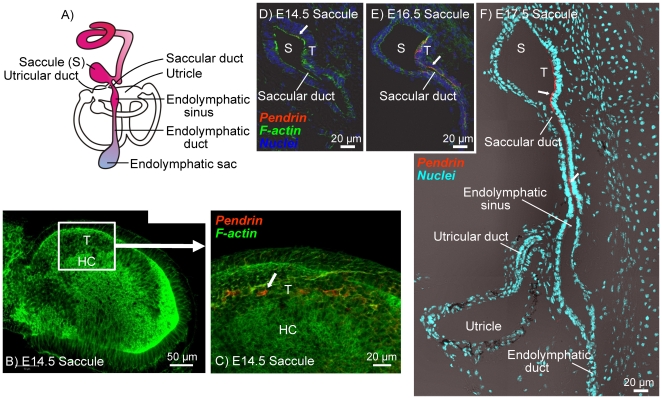
Onset of pendrin expression in the saccule of the vestibular labyrinth. Pendrin (*red*) was visualized by immunocytochemistry. F-actin (*green*) and nuclei (*blue*) were labeled. A: Diagram of the inner ear. B–C: Whole-mount of the saccule from a *Slc26a4^+/−^* mice at age E14.5. D–F: Cross-sections of the saccule from *Slc26a4^+/−^* mice at age E14.5–E17.5. Abbreviations: S, Saccule; T, transitional cells; HC, hair cells. Pendrin expression is marked by arrows.

In the vestibular labyrinth, pendrin expression was found in transitional cells surrounding the sensory cells in the utricle, saccule and semicircular canal ampullae, as well as in epithelial cells that line the saccular duct. The onset of pendrin expression in the anterior semicircular canal ampulla was at E16.5 (Fig. 2A–D), which is two days after the onset of expression in utricular transitional cells, which occurred at E14.5 (Fig. 2E–I). In the saccule, the onset of pendrin expression in transitional cells was at E14.5 (Fig. 3A–C) and in cells lining the saccular duct was at E15.5–E16.5 (Fig. 3D–F).

**Figure 4:**
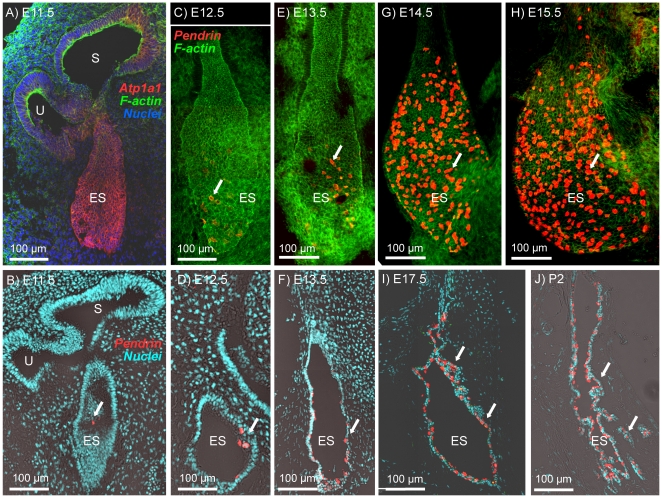
Onset of pendrin expression in the endolymphatic sac. Na^+^/K^+^ ATPase or pendrin (*red*) were visualized by immunocytochemistry. F-actin (*green*) and nuclei (*blue*) were labeled. A, B, D, F, I and J: Cross-sections of the endolymphatic sac from *Slc26a4^+/−^* mice at ages E11.5-P2. C, E, G and H: Whole-mounts of the endolymphatic sac from *Slc26a4^+/−^* mice at age E12.5–E15.5. Abbreviations: U, utricle; S, saccule; ES, endolymphatic sac. Pendrin expression is marked by arrows.

The endolymphatic sac at E10.5 was identified as a pouch-like structure composed of epithelial cells expressing Na^+^/K^+^ ATPase that projected from the dorso-medial side of the otocyst opposite to the cochlea duct that emerged at the ventral side (Fig. 4A). A fluid-filled lumen was formed in the endolymphatic sac at E11.5–E12.5 (Fig. 4B and D). The onset of pendrin expression was at E11.5 (Fig. 4B). The number of cells expressing pendrin increased with development (Fig. 4B–J). The overall organ size grew notably from E13.5 to E14.5 (Fig. 4E and G). From E14.5 onward, almost 30% of cells in the distal portion of endolymphatic sac expressed pendrin (Fig. 4G and H). At E17.5 and P2, the endolymphatic sac epithelium became increasingly rugous with infoldings and tubular protrusions (Fig. 4I and J).

### Onset of pendrin-dependent pH changes

**Figure 5:**
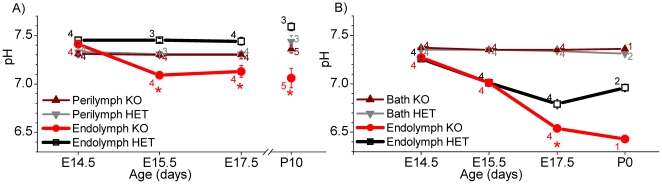
pH measurements in the embryonic inner ear. A: Measurements of the endolymphatic and perilymphatic pH in the cochlea of isolated *in vitro* superfused otocysts from *Slc26a4^+/−^* (HET) and *Slc26a4^−/−^* (KO) mice aged E14.5–E17.5. Data from P10 *Slc26a4^+/−^* mice, obtained by *in situ* measurements and reported earlier [11], are shown here for comparison. B: Measurements of the endolymphatic pH in the endolymphatic sac of isolated otocysts from *Slc26a4^+/−^* (HET) and *Slc26a4^−/−^* (KO) mice aged E14.5–E17.5. Numbers next to the bars represent the N number of otocysts. Significant differences between endolymph from *Slc26a4^+/−^* (*black*) and *Slc26a4^−/−^* (*red*) mice are marked with a star.

In a second series of experiments, we measured in isolated *in vitro* superfused otocysts the transepithelial potential and the pH of endolymph and perilymph in the hook region of the cochlea. Further, we measured the transepithelial potential and the pH of endolymph in the endolymphatic sac. Measurements were made in *Slc26a4^+/−^* and *Slc26a4^−/−^* littermates ranging from age E14.5 to E17.5. The transepithelial potential in the cochlea and the endolymphatic sac was near zero at all ages and genotypes (*data not shown*). The perilymphatic pH in the cochlea was 7.32±0.05 (SD; n = 15) and did not vary with development or genotype (Fig. 5A). The endolymphatic pH was dependent on the age and genotype of the embryo. At E14.5, the pH of cochlear endolymph was by ∼0.1 pH-units larger than the pH of perilymph with no difference between *Slc26a4^+/−^* and *Slc26a4^−/−^* mice. At E15.5 and E17.5 the pH of cochlear endolymph in *Slc26a4^+/−^* mice remained by ∼0.1-pH-units more alkaline than the pH of perilymph but the endolymphatic pH in *Slc26a4^−/−^* became ∼0.3 pH-units more acid than the endolymphatic pH in *Slc26a4^+/−^* mice.

The pH of the bath fluid surrounding the endolymphatic sac was 7.35±0.04 (SD; n = 25). At E14.5 the pH in the endolymphatic sac of *Slc26a4^+/−^* and *Slc26a4^−/−^* mice was ∼0.1 pH-units lower than the pH of the bath solution (Fig. 5B). At E15.5 and E17.5 the endolymphatic pH in *Slc26a4^+/−^* mice dropped to ∼0.3 and ∼0.5 pH-units below the pH of the bath fluid, respectively. No differences were observed between *Slc26a4^+/−^* and *Slc26a4^−/−^* mice at E14.5 and E15.5, however, at E17.5 the endolymphatic pH in *Slc26a4^−/−^* mice was ∼0.8 pH-units more acidic than the pH of the bath solution and ∼0.2 pH-units more acid than endolymphatic pH in *Slc26a4^+/−^* mice.

### Pendrin-dependent luminal enlargement and epithelial cell stretching

**Figure 6:**
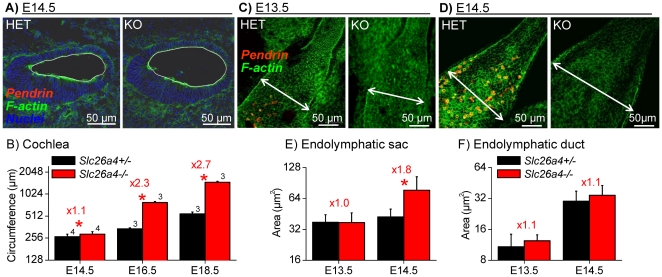
Onset of cell stretching in cochlea and endolymphatic sac. Pendrin (*red*) was visualized by immunocytochemistry. F-actin (*green*) and nuclei (*blue*) were labeled. A: Cross-sections of the basal turn of the cochlea from *Slc26a4^+/−^* (HET) and *Slc26a4^−/−^* (KO) mice at age E14.5. The circumference of the cochlear lumen is marked by a white line. B: Summarized measurements (avg±sem) of the luminal circumference of the cochlea at ages E14.5–E18.5. Numbers next to the bars represent the N number of otocysts. Significant differences between *Slc26a4^+/−^* (*black*) and *Slc26a4^−/−^* (*red*) mice are marked with a star. C–D: Whole-mounts of the endolymphatic sac from *Slc26a4^+/−^* (HET) and *Slc26a4^−/−^* (KO) mice at ages E13.5 and E14.5. The width of the endolymphatic sac is marked by a double-arrow. E–F: Summarized measurements (avg±SD, each bar N = 15) of the apical surface area of epithelial cells in the endolymphatic sac and duct at ages E13.5–E14.5. Significant differences between *Slc26a4^+/−^* (*black*) and *Slc26a4^−/−^* (*red*) mice are marked with a star. Figures preceded by ‘x’ indicate the factor between measurements in *Slc26a4^+/−^* and *Slc26a4^−/−^* mice.

In a third series of experiments, we evaluated epithelial cell stretching by measuring the circumference of the cochlear lumen and by measuring the apical surface areas of individual epithelial cells. Measurements of the circumference of the cochlea lumen were made in matched cross-sections of the basal turn of *Slc26a4^+/−^*and *Slc26a4^−/−^* littermates (Fig. 6A–B). At E14.5 the length of the circumference of the cochlear lumen was larger in *Slc26a4^−/−^* mice compared to *Slc26a4^+/−^* mice, although the increase in length was not substantial, factor 1.1. With further development the difference in length of the circumference between *Slc26a4^+/−^* and *Slc26a4^−/−^* mice grew from a factor of 1.1 to a factor of 2.3 at E16.5 and 2.7 at E18.5 (Fig. 6B).

The overall organ size of the endolymphatic sac at E13.5 was similar between *Slc26a4^+/−^* and *Slc26a4^−/−^* mice, however, one day later, at E14.5, the endolymphatic sac was notably enlarged in *Slc26a4^−/−^* mice (Fig. 6C–D). Consistently, apical cell surface areas in the endolymphatic sac were similar at E13.5 but enlarged at E14.5 (Fig. 6E), however, apical cells surface areas in the endolymphatic duct remained similar between *Slc26a4^+/−^* and *Slc26a4^−/−^* mice at E13.5 and at E14.5 (Fig. 6F).

**Figure 7:**
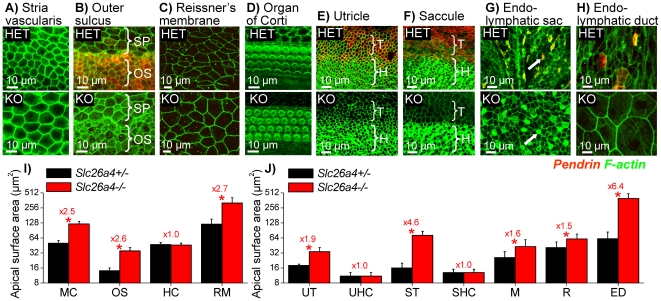
Epithelial cell stretching in the cochlea and the vestibular labyrinth at P2. Pendrin (*red*) was visualized by immunocytochemistry and F-actin (*green*) was labeled in *Slc26a4^+/−^* (HET) mice and *Slc26a4^−/−^* (KO) mice. A–D: Whole-mounts of epithelial cells from the cochlea. Abbreviations: SP, spiral prominence epithelial cells; OS, outer sulcus epithelial cells. E–H) Whole-mounts of epithelial cells from the vestibular labyrinth and endolymphatic sac. Mitochondria-rich cells in endolymphatic sac from *Slc26a4^+/−^* and *Slc26a4^−/−^* mice are marked by arrows. Abbreviations: T, transitional cells; H, hair cells. I: Summary of apical cell surface area measurements (avg±SD, N = 15) made on cochlea epithelial cells. Abbreviations: MC, marginal cells of stria vascularis; OS, outer sulcus epithelial cells; HC, hair cells; RM, Reissner's membrane epithelial cells. J: Summary of apical cell surface area measurements (avg±SD, N = 15) made on vestibular epithelial cells. Abbreviations: UT, utricular transitional cells; UHC, utricular hair cells; ST, saccular transitional cells; SHC, saccular hair cells; M, mitochondria-rich cells; R, ribosome-rich cells; ED, endolymphatic duct epithelial cells. Significant differences between *Slc26a4^+/−^* (*black*) and *Slc26a4^−/−^* (*red*) mice are marked with a star. Figures preceded by ‘x’ indicate the factor between measurements in *Slc26a4^+/−^* and *Slc26a4^−/−^* mice.

Stretching of epithelial cells in the cochlea, the vestibular labyrinth and the endolymphatic sac and duct was evaluated in greater detail by measuring apical surface areas in *Slc26a4^+/−^* and *Slc26a4^−/−^* mice at age P2 (Fig. 7). In the cochlea, apical surface areas of strial marginal cells, outer sulcus epithelial cells, and Reissner's membrane epithelial cells were enlarged in *Slc26a4^−/−^* mice compared to *Slc26a4^+/−^* mice (Fig. 7A–C and I). In contrast, apical surface areas of inner and outer hair cells were similar and not different between *Slc26a4^+/−^* and *Slc26a4^−/−^* mice (Fig. 7D and I). In the vestibular labyrinth, apical surface areas of transitional cells in the utricle and saccule were enlarged in *Slc26a4^−/−^* mice compared to *Slc26a4^+/−^* mice, however, similar to the cochlea, apical surface areas of hair cells did not differ between *Slc26a4^+/−^* and *Slc26a4^−/−^* mice (Fig. 7E–F and J). The epithelium of the endolymphatic sac in *Slc26a4^+/−^* was heavily folded and included tubular protrusions, as shown in Fig. 4J. In contrast, the epithelium in *Slc26a4^−/−^* mice was planar due to cell stretching caused by the enlargement. Mitochondria-rich cells that expressed pendrin in *Slc26a4^+/−^* mice were distinguished from ribosome-rich cells in *Slc26a4^+/−^* and *Slc26a4^−/−^* mice by their dense actin expression near the apical membrane (Fig. 7G). Cells oriented perpendicular to the optical path were selected for apical surface area measurements in *Slc26a4^+/−^* and *Slc26a4^−/−^* mice. Apical surface areas of mitochondria-rich and ribosome-rich cells were found to be enlarged in *Slc26a4^−/−^* mice compared to *Slc26a4^+/−^* mice (Fig. 7J). Further, apical surface areas of epithelial cells lining the endolymphatic duct were found to be enlarged in *Slc26a4^−/−^* mice compared to *Slc26a4^+/−^* mice (Fig. 7H and J).

**Figure 8:**
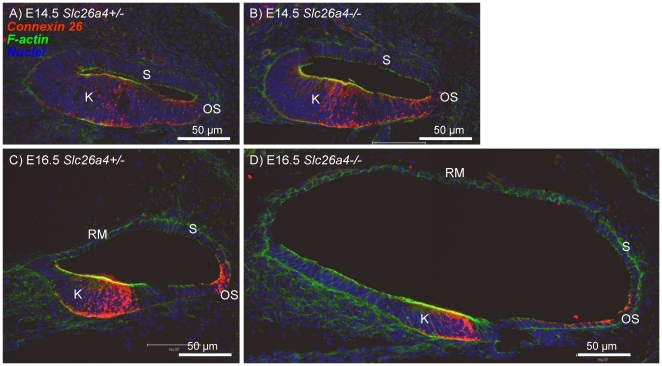
Connexin 26 expression in the cochlea of *Slc26a4* ^+/−^ and *Slc26a4* ^−/−^ mice at E14.5 and E16.5. Connexin 26 (*red*) was visualized by immunocytochemistry. F-actin (*green*) and nuclei (*blue*) were labeled. A–B: Cross-sections of the basal turn of the cochlea from *Slc26a4^+/−^* and *Slc26a4^−/−^* mice at age E14.5. C–D: Cross-sections of the basal turn of the cochlea from *Slc26a4^+/−^* and *Slc26a4^−/−^* mice at age E16.5. Abbreviations: K, Kölliker's organ; RM, Reissner's membrane; S, stria vascularis; OS, outer sulcus.

**Figure 9:**
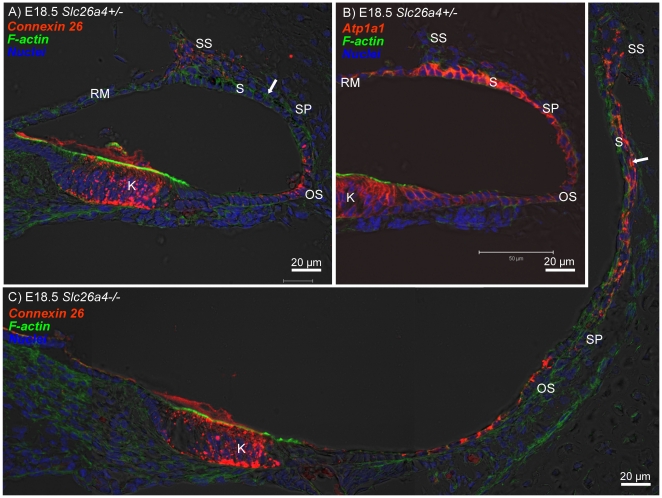
Connexin 26 and Na^+^/K^+^ ATPase expression in the cochlea of *Slc26a4* ^+/−^ and *Slc26a4* ^−/−^ mice at E18.5. Connexin 26 and Na^+^/K^+^ ATPase (both *red*) were visualized by immunocytochemistry. F-actin (*green*) and nuclei (*blue*) were labeled. A–B: Cross-sections of the basal turn of the cochlea from *Slc26a4^+/−^* mice at age E18.5. C: Cross-sections of the basal turn of the cochlea from *Slc26a4^−/−^* mice at age E18.5. Abbreviations: K, Kölliker's organ; RM, Reissner's membrane; SS, fibrocytes in the suprastrial region; S, stria vascularis; SP, spiral prominence; OS, outer sulcus. The arrows in A and C point to basal cells at E18.5 that lack connexin 26 expression in *Slc26a4^+/−^* mice but express connexin 26 prematurely in *Slc26a4^−/−^* mice.

**Figure 10:**
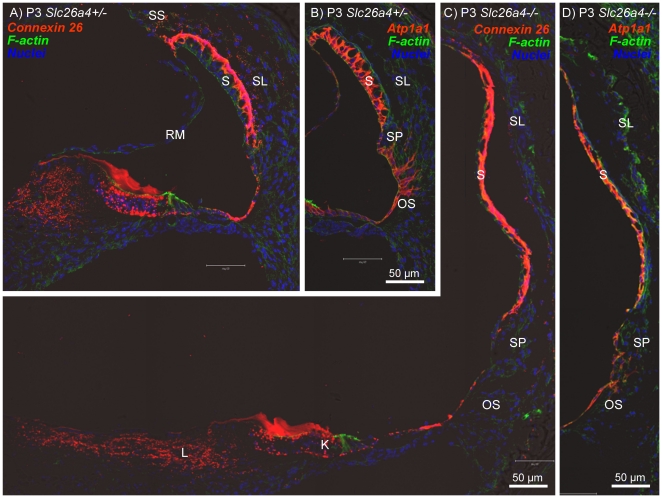
Connexin 26 and Na^+^/K^+^ ATPase expression in the cochlea of *Slc26a4* ^+/−^ and *Slc26a4* ^−/−^ mice at P3. Connexin 26 and Na^+^/K^+^ ATPase (both *red*) were visualized by immunocytochemistry. F-actin (*green*) and nuclei (*blue*) were labeled. A–B: Cross-sections of the basal turn of the cochlea from *Slc26a4^+/−^* mice at age P3. C–D: Cross-sections of the basal turn of the cochlea from *Slc26a4^−/−^* mice at age P3. Abbreviations: K, Kölliker's organ; L, fibrocytes in the spiral limbus; RM, Reissner's membrane; SS, fibrocytes in the suprastrial region; S, stria vascularis; SL, spiral ligament; SP, spiral prominence; OS, outer sulcus.

In a fourth series of experiments, we evaluated expression patterns of connexin 26 and Na^+^/K^+^ ATPase in the cochlea of *Slc26a4^+/−^* and *Slc26a4^−/−^* mice. At E14.5, connexin 26 was expressed in gap junction plaques in Kölliker's organ and in outer sulcus epithelial cells. The expression pattern at this age was similar in *Slc26a4^+/−^* and *Slc26a4^−/−^* mice (Fig. 8A and B). At E16.5, the region of connexin 26 expression in Kölliker's organ became distinct from the region of in outer sulcus epithelial cells (Fig. 8C and D). Cell height of outer sulcus epithelial cells was lower in *Slc26a4^−/−^* mice compared to *Slc26a4^+/−^* mice. This thinning of the epithelium is most likely due to stretching. The stretch-related reduction in the area of lateral approximation between epithelial cells was reflected in the staining pattern of connexin 26 that appeared concentrated toward the tight-junction complexes. At E18.5, connexin 26 expression was found in *Slc26a4^+/−^* and *Slc26a4^−/−^* mice in Kölliker's organ, in outer sulcus epithelial cells and in fibrocytes of the suprastrial region (Fig. 9A and C). Expression of Na^+^/K^+^ ATPase was found in all epithelial lining the cochlea lumen (Fig. 9B). The highest intensity of Na^+^/K^+^ ATPase expression was found in stria vascularis. At P3, intense expressions of connexin 26 in basal cells and of Na^+^/K^+^ ATPase in marginal cells of stria vascularis were found in *Slc26a4^+/−^* and *Slc26a4^−/−^* mice (Fig. 10A–D).

Tissue height of stria vascularis was much lower in *Slc26a4^−/−^* mice compared to *Slc26a4^+/−^* mice. This thinning of the tissue is most likely due to stretching. Interestingly, the onset of connexin 26 expression in basal cell of stria vascularis appeared earlier in *Slc26a4^−/−^* mice compared to *Slc26a4^+/−^* mice: Expression was found at E18.5 in *Slc26a4^−/−^* mice but not yet in *Slc26a4^+/−^* mice (Fig. 9A and C). At P3, the intensity of expression appeared similar in *Slc26a4^+/−^* and *Slc26a4^−/−^* mice (Fig. 10A and C).

**Figure 11:**
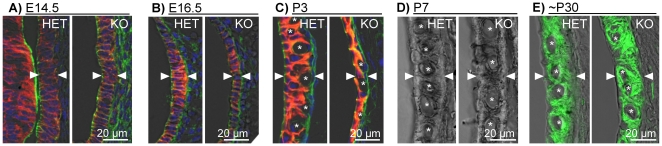
Retarded development of stria vascularis. A–C: Cross-sections of stria vascularis of *Slc26a4^+/−^* (HET) and *Slc26a4^−/−^* (KO) mice at E14.5, E16.5 and P3. Na^+^/K^+^ ATPase (*red*) was visualized by immunocytochemistry. F-actin (gr*e*en) and nuclei (*blue*) were labeled. D: Cross-sections of stria vascularis at P7 visualized by laser-scanning microscopy without any stain. E: Cross-sections of stria vascularis at ∼P30. The Na^+^/2Cl^−^/K^+^ cotransporter *Slc12a2* was visualized by immunocytochemistry. The ∼P30 data, previously reported [16], were added here for comparison. Tissue height of stria vascularis is marked by arrow-heads. Cross-sections of individual capillaries are marked with a star.

The development of stria vascularis was evaluated between E14.5 and P7 (Fig. 11). The expression pattern of Na^+^/K^+^ ATPase was similar between *Slc26a4^+/−^* and *Slc26a4^−/−^* mice, however, from E16.5 to P3, tissue height of stria vascularis was lower in *Slc26a4^−/−^* mice. At P3, stria vascularis became visibly endowed with a network of capillaries (Fig. 11C). The diameter of individual capillaries was smaller in *Slc26a4^−/−^* mice and the capillary network appeared less dense compared to *Slc26a4^+/−^* mice. At P7, tissue height of stria vascularis appeared similar in *Slc26a4^+/−^* and *Slc26a4^−/−^* mice (Fig. 11D). Diameters of individual capillaries appeared similar at this age, however, the density of the capillary network remained less, which is consistent with tissue stretching.

## Discussion

The most salient findings of the present study are: 1) expression of pendrin in the inner ear begins in the endolymphatic sac followed by the onset of expression in the cochlea and vestibular labyrinth. 2) failure to express pendrin causes acidification of the luminal fluid in the cochlea and the endolymphatic sac and that this acidification occurs in the cochlea within 1–2 days of the failed onset of expression and in the endolymphatic sac after more than 4 days after the failed onset. 3) failure to express pendrin causes an enlargement of the endolymphatic sac that develops within 3 days of the failed onset of expression, whereas the enlargement of the cochlea precedes the onset of expression by ∼1 day. 4) failure to express pendrin causes stretching of non-sensory epithelial cells and a retarded development of stria vascularis.

### Pendrin protein expression

The expression pattern of pendrin in the developing inner ear is consistent with the pattern that had previously been determined in the adult inner ear [15], [16]. Accordingly, pendrin expression was found in the developing cochlea in outer sulcus epithelial cells, in transitional cells of the developing semicircular canal ampullae, utricle and saccule as well as in the developing endolymphatic sac (Fig. 1, 2, 3, 4). An additional location of expression was the epithelium that lines the saccular duct (Fig. 3). The role of this epithelium in inner ear fluid homeostasis is unknown.

The observation that pendrin protein expression was found to occur first in the endolymphatic sac is consistent with earlier investigations monitoring the onset of pendrin mRNA expression [24]. Our data document the onset of expression at E11.5, which is 2 days prior to the earlier report that was based on *in situ* hybridization. This difference is important, because the onset of pendrin expression in the endolymphatic sac at E11.5 coincides with the onset of H^+^ ATPase expression [25]. H^+^ ATPase, like pendrin, is expressed in the apical membrane of mitochondrial-rich cells in the endolymphatic sac. The coincidental onset of expression suggests that both transporters form a functional unit that transports HCO_3_^−^ buffered H^+^ across the apical membrane.

### Failure to express pendrin causes luminal pH changes

We have previously shown in the adult cochlea that the endolymphatic pH is higher than the perilymphatic pH when pendrin is expressed and lower than the perilymphatic pH when pendrin is absent [11]. Similar observations have now been made in the developing cochlea as early as E15.5, which is 1–2 days after the onset of pendrin expression in the cochlea (Fig. 5). Interestingly, one day earlier, at E14.5, the endolymphatic pH was by ∼0.1 pH-units higher than the perilymphatic pH regardless of the absence of presence of pendrin. This observation suggests the presence of other acid or base transport systems that contribute to the elevated pH of endolymph at least at this stage of development.

In contrast to the endolymphatic pH in the cochlea, the pH of the endolymphatic sac was found to be more acidic than the surrounding fluid. This acidic pH was observed as early as E14.5, which is 3 days after the onset of pendrin and H^+^ ATPase expression [25] (Fig. 5). The finding of an acidic pH in the developing endolymphatic sac is consistent with measurements in the adult endolymphatic sac [26], [27], [28]. Interestingly, up to E15.5, which is 4 days after the onset of pendrin expression, no difference in the endolymphatic pH was found between *Slc26a4^+/−^* and *Slc26a4^−/−^* mice. It remains undetermined, whether this delay in luminal pH changes is due to compensatory mechanisms possibly consisting of a coupling between the rates of ATPase-mediated H^+^ secretion and pendrin-mediated HCO_3_^−^ secretion. Alternatively, the delay in the appearance of luminal pH changes could be related to the enlarged luminal volume found in *Slc26a4^−/−^* mice or simply be a function of luminal pH buffering. Buffering of the luminal pH can be expected to be stronger in the endolymphatic sac compared to the cochlea since the endolymphatic sac, at least in adult animals, contains a higher concentration of proteins [29], [30], [31].

### Failure to express pendrin causes stretching and retarded development of epithelial cells

We have previously reported that lumen formation in the embryonic cochlea is controlled by fluid secretion in the vestibular labyrinth and fluid absorption in the endolymphatic sac and that fluid absorption in the endolymphatic sac depends on pendrin [14]. The observation that the onset of the cochlear enlargement precedes the onset of pendrin expression (Fig. 6) supports the concept that the enlargement is initially driven by fluid secretion in the vestibular labyrinth rather than by defect of local cochlear fluid transport. Nothing is known about ion and fluid transport in the embryonic vestibular labyrinth, although the observation that the endolymphatic sac forms an open lumen at E11.5–E12.5 suggests that fluid secretion in the vestibular labyrinth is present at this time. It is an attractive hypothesis to assume that the cochlea and the endolymphatic sac, which both form as pouches protruding from the otocyst, are ‘pumped up’ by fluid secretion in the otocyst that forms the vestibular labyrinth. The observation that the onset of the enlargement of the cochlea and the onset of the enlargement of the endolymphatic sac coincide at E14.5 (Fig. 6), is consistent with the hypothesis of a common source of fluid secretion.

The role of the vestibular labyrinth in the control of the cochlear lumen is limited to the phase of development prior to E16.5, since the utricular duct, which connects the utricle to the saccule, is closed after E16.5 [32]. Closing of the utricular duct creates two independent fluid systems. One system consisting of the cochlea, saccule and endolymphatic sac and the other consisting of the utricle, ampullae and semicircular canals [32]. The observation that the cochlear lumen continues to increase after E16.5 suggests the presence of a local defect in fluid homeostasis leading to abnormally high rates of fluid secretion or abnormally low rates of fluid reabsorption [14]. Abnormally low rates of fluid absorption are conceivably due to an impairment of Na^+^ reabsorption in Reissner's membrane epithelial cells. Reissner's membrane epithelial cells, analogous to principal cells in the renal cortical collecting duct, are engaged in Na^+^ reabsorption via ENaC Na^+^ channels and face a fluid compartment that is controlled in its pH and HCO_3_^−^ concentration by pendrin [33], [34]. Recent observations in the renal collecting duct suggest that a lower HCO_3_^−^ concentration reduces the abundance of ENaC Na^+^ channel subunits [35]. How this mechanism works and whether it is present in the inner ear is unknown.

The enlargement of the cochlear lumen is clearly a key event in the etiology of deafness in mice lacking pendrin [12], [14]. The enlargement impairs cell communication between mesenchymal and epithelial cells and causes stretching of epithelial cells. Stretching lengthens diffusional distances and thereby may impair cell-to-cell communication among epithelial cells. Interestingly, non-sensory cells are stretched but sensory cells are not (Fig. 7). This difference is most likely the result of differences in the epithelial rigidity. Sensory cells are endowed with a thick actin-ring near their apical tight junctions, which may provide the sensory epithelia with a greater rigidity which limits cell stretching to the more compliant non-sensory cells.

The enlargement and the acidification spread the effect of lacking pendrin-expression from affecting just pendrin-expressing cells to affecting a multitude of other cells. Expression patterns of connexin 26 and Na^+^/K^+^ ATPase in Kölliker's organ and the outer sulcus region appeared qualitatively similar in *Slc26a4^+/−^* and *Slc26a4^−/−^* mice (Fig. 8, 9, 10). However, the onset of connexin 26 expression in basal cells of stria vascularis occurred in *Slc26a4^−/−^* prematurely (Fig. 9) and the expression pattern of connexin 26 and Na^+^/K^+^ ATPase highlighted stretching and thinning of stria vascularis (Fig. 10). The discrepancy in development of stria vascularis between *Slc26a4^+/−^* and *Slc26a4^−/−^* mice was most apparent at P3 (Fig. 11). The observation that loss of pendrin leads to a retarded development of stria vascularis including a retarded vascularization is consistent with an impairment of cell-to-cell communication. Structural differences between stria vascularis of *Slc26a4^+/−^* and *Slc26a4^−/−^* mice diminished at P7, however, oxidative stress and a reduced endocochlear potential have been found in *Slc26a4^−/−^* mice at P10 and stria vascularis undergoes degeneration at P30 including an invasion of macrophages [36], [37], [11]. Taken together, these observations demonstrate that the retarded development of stria vascularis precludes normal function of stria vascularis in the postnatal cochlea.

In summary, the present study documents the onset of pendrin expression and the onset of functional consequence resulting from a failed onset of expression. Lack of pendrin expression leads to epithelial cell stretching and luminal acidification, which may alter cell signaling to result in the observed retarded development of stria vascularis. Retarded development of stria vascularis and possibly of other non-sensory epithelial cells may be an important step on the path to deafness in *Slc26a4^−/−^* mice, and possibly humans, lacking functional pendrin expression.
